# Visit from Dr. Cartwright

**Published:** 1848-02

**Authors:** 


A VISIT FROM DR. CARTWRIGHT.—MEDICAL STATISTICS.—EPIDEMIC CHOLERA.

We lately had a visit from the distinguished physician, and able medical writer, Dr. Samuel A. Cartwright, of Natchez, while on a tour of observation and improvement of health. On the invitation of the medical class of our University, Dr. Cartwright delivered before them two lectures on medical statistics, and the numeral method of testing the effects of different plans of treatment; in the course of which he pointed out several of the efficient causes of the great prevalence of empiricism, and the vast consumption of nostrums, patented and unpatented, by the people of the south and west. He announced as the result of his statistical references, that human life, in the aggregate, is not so long in the valley, as it is east of the mountains; and assigns, as one of the causes, the overweening reliance of the people on empirical remedies, with the gross adulteration of many of the more important medicines, sold to our physicians and retail apothecaries. In reference to the latter he stated, as a fact, that medicine chests are sent into the southwest, containing the medicines of the shops, and certain preparations of them, under various empirical names. The former he had ascertained to be often adulterated or effete, while the latter were composed of genuine and active articles. As examples, he mentioned sulphate of quinine, calomel, aperient pills, and vermifuges. The quinine is often mixed with cinchonine, the calomel contains corrosive sublimate, the aloes sold, are the hepatic, while the quacks, and eastern druggists in collusion with them, monopolize the socotorine, and work it up into cathartic pills; the recent spigelia is monopolized in the same way, and the wormseed oil, sold to our apothecaries, is adulterated, while the genuine and active is labelled as a nostrum! All this is truly deplorable, but what or where is the remedy?

We asked of the Doctor his first lecture for publication in our journal, and the class made the same request. We are happy to say, that it will appear in our next number, and, simultaneously, as a separate publication, by the class.

As the professor of Pathology and Practice, happened to be lecturing on epidemic cholera, at the time of the Doctor's visit, by request of the former, he came before the class and delivered two extempore lectures, on the history and treatment of that disease at Natchez, in 1832. It will be recollected by many of our readers, that the Doctor's great remedy, was the following compound:

R. Powderd Capsicum, grs. xx.
Calomel, grs. xx.
Camphor, grs. x.—Mix.

To be taken, either in powder or pills, every two, three, or four hours; and according to the testimony of many respectable planters at the time, and the declarations of the Doctor in his lectures, this remedy was eminently successful. In the constitutions of the people further north, it was not, however, on the whole, as effective as the compound of calomel and opium, which made the basis of all successful treatment.

We have reproduced these facts, at this time, because of the threatened second invasion of the same epidemic. That the disease has, for the last year or two, been advancing to the west of Asia, and several months since entered Europe, seems quite certain. How long it will be in traversing that continent, we cannot say, but recent (though not reliable) intelligence, represents it as having reached London. If so, our readers will have the account confirmed to them, through the newspapers, before they will see this article. But whether true or false, every physician should expect a visitation at no distant time, and prepare himself to meet it, by a full review of the various methods of treatment, pursued in the last. The medical population changes so fast in this country, that at least one-half of our present physicians, are unacquainted, except from reading or lectures, with the epidemic of 1832–'4. It behooves all such, to apply themselves diligently to the study of what was then and afterwards published; that the invader may not find them either sleeping, or unfurnished with the best means of defence.

